# Supplementary material for: Alleviating Effect of α-Lipoic Acid and Magnesium on Cadmium-Induced Inflammatory Processes, Oxidative Stress and Bone Metabolism Disorders in Wistar Rats
Source: Int J Environ Res Public Health. 2019 Nov 14;16(22):4483. doi: 10.3390/ijerph16224483 (PMC6888481; doi:10.3390/ijerph16224483)
Supplement: Supplementary file 1 [file ijerph-16-04483-s001.pdf]

Effect of cadmium treatment and supplementation with Mg and/or **α-LA** on the Absolute Kidney Mass (AKM), Relative Liver Mass (RLM) and Relative Liver Mass (RLM)

	**Control**	**Cd**	**Cd+α-LA**	**Cd+Mg**	**Cd+Mg+α-LA**
AKM [mg]	1.33(±0.21)	1.30(±0.13)	1.35(±0.07)	1.22(±0.11)	1.26(±0.11)
RKM	0.32(±0.03)	0.34(±0.02)	0.40(±0.04)[ab]	0.37(±0.05) [a]	0.35(±0.02)[c]
RLM	3.02(±0.32)	3.26(±0.20)	3.58(±0.22) [ab]	3.45(±0.13) [a]	3.46(±0.28) [a]

Explanation:

AKM - Absolute Kidney Mass. RLM. RKM - relative liver, kidney mass = organ/body mass x100.

[1] Values are expressed as mean (± SD); [a] Significant change from control (C). $p < 0.05$; [b] Groups exposed to Cd separately significantly different from group exposed to Cd and supplemented with Mg and/or α-lipoic (Cd vs. Cd+α-LA or Cd vs. Cd+Mg or Cd vs. Cd+Mg+α-LA). $p < 0.05$; [c] Cadmium groups supplemented with α-lipoic acid significantly different from cadmium groups supplemented with Mg and cadmium group co-supplemented with Mg and α-lipoic acid (Cd+α-LA vs. Cd+Mg or Cd+α-LA vs. Cd+Mg+α-LA). $p < 0.05$; [d] Cadmium group supplemented only with Mg significantly different from cadmium group co-supplemented with Mg and α-lipoic acid (Cd+Mg vs. Cd+Mg+α-LA). $p < 0.05$.

The rats were under constant observation of the laboratory workers. No worrying signs in behavior, activity, or appearance were observed during the experiment. No worrying clinical signs were found such as diarrhea, hair bristling, cage soaking, pale eyes, or distended abdomen (the observation forms were filled in daily). During the autopsy, a macroscopic evaluation of the organs was performed. No changes were observed in the shape, size, color, or consistency of liver or kidneys in the cadmium-poisoned groups as compared with the organs in the control group. The livers and kidneys were weighed. Relative mass was calculated for liver and kidney. Moreover, absolute mass was compared for kidneys (as more reliable than RKM for identifying potential renal toxicants [1]**.**

The differences in the absolute mass of kidneys across the rat groups were not statistically significant. Higher RLM and RKM was observed in Cd groups supplemented with Mg and/or α-lipoic in relation to the control group. Among all groups of rats, the highest relative liver and kidney mass was reported in groups supplemented with α-lipoic acid separately. However, owing to lower values of liver enzymes activity and better biochemical parameters (in comparison to the Cd group), these changes were considered as an adaptive response of the organ to increased functional load associated with the metabolism of xenobiotics and supplement.

[1] *Craig E.A. et al. The relationship between chemical-induced kidney weight increases and kidney histopathology in rats J. Appl. Toxicol. 2015; 35: 729–736.*
